# Publication Activity and Impact of the International Pediatric Simulation Society Cureus Channel: 2014 to 2018

**DOI:** 10.7759/cureus.4067

**Published:** 2019-02-13

**Authors:** Taylor Sawyer, Edward J. Rovera

**Affiliations:** 1 Pediatrics, University of Washington School of Medicine, Seattle, USA; 2 Medical Education and Simulation, San Francisco State University, San Francisco, USA

**Keywords:** bibliometrics, scientometrics, simulation, professional development, scholarship, pediatrics, pediatric simulation

## Abstract

Introduction

In November 2013, the International Pediatric Simulation Society (IPSS) launched a channel on Cureus.com. We performed the following analysis to examine the use of the channel, the reach of its publications, and their impact within the medical literature.

Methods

The IPSS Cureus channel’s Administrative Dashboard was used to collect data on manuscript type, manuscript submission date, publication date, total views, and total downloads for each article from publication date through December 31, 2018. Web of Science and Google Scholar were used to determine which channel publications had been cited during the study period, and those citations were reviewed to develop an estimate of the global reach of the publications.

Results

A total of 15 articles were published via the IPSS Cureus channel from April 18, 2014 to December 31, 2018, with a mean time between submission and publication of 46 days. These articles have been viewed an average of 736 times per article. The PDF download rate averaged 446 per article. These 15 articles have been cited a total of 37 times, averaging 2.5 citations per article. The sources for those citations were published in English (29), Spanish (4), Portuguese (2), Arabic (1), and Ukrainian (1).

Conclusion

Publications on the IPSS Cureus channel have a clear impact, in terms of the number of views, number of downloads, citation counts, and global reach. The channel also offers a rapid publication cycle. Further education in the pediatric simulation community on the use of the channel and promotion of the benefits of this resource for scholarships are warranted.

## Introduction

In 2012, the International Pediatric Simulation Society (IPSS, the "Society") began discussions with Cureus.com, to develop a portal allowing IPSS members to publish their pediatric simulation work. Since 2014, IPSS members have been able to publish their work on the IPSS Cureus channel (www.cureus.com/channels/ipss). Selected Society members perform an editorial review on all articles submitted through the Society’s channel. This service is provided at no cost to IPSS members as Cureus has no associated publication fees.

All publications on the IPSS Cureus channel are indexed in PubMed Central, the largest repository of biomedical literature in the world [1]. In turn, PubMed Central is itself indexed in Web of Science (WOS), one of the two largest multi-discipline repositories of scholarly data currently in existence [2].

In order to better understand the usage of the channel, the authors conducted the following study to examine the publication activity and impact of the articles published on the channel. Our specific aim was to attempt to answer the question: What are the publication activity and scholarly impact of the IPSS Cureus channel?

## Materials and methods

Publication activity on the IPSS Cureus channel was analyzed using the channel’s Administrative Dashboard. Data on views and downloads were collected from April 18, 2014 to December 31, 2018 (55 months). Manuscript type, manuscript submission date, publication date, total views, and total downloads for each article were analyzed. Since the longer an article was able to be viewed or downloaded would increase its chances of being viewed or downloaded, the number of views and downloads was measured against the length of time the article had been published in order to give a more accurate measurement of the interest generated by each publication.

The Administrative Dashboard was used to perform an analysis of the IPSS Cureus channel’s publication cycle. The dashboard provides the dates of all events pertaining to the publication cycle of any manuscript. The overall length of time between submission and final publication was determined, as were the time points between key steps in the process. The total time from submission to publication was broken into four distinct phases:

1. First editorial review: Once the submitting author requests a manuscript to go into Peer Review, the IPSS Cureus channel editors and one of the Cureus editorial staff perform separate editorial reviews in sequence. The manuscript must pass both editorial reviews to be moved forward to Peer Review.

2. Peer review: This is the length of time the manuscript is being reviewed by members of the Cureus community and the authors are responding to the recommendations of the reviewers. When the authors feel they have answered the reviewers’ questions, the submitting author requests publication.

3. Second editorial review*:* Prior to publication, the IPSS Cureus channel editors and the Cureus editor again review the manuscript, this time focusing on the reviewers’ comments and the authors’ responses. Both also make a final copy edit to meet the requirements of the PubMed Central indexing service. Again, the manuscript must pass both editorial reviews to be approved for publication.

4. Publication process: When a manuscript has been approved for publication, a final migration from the Cureus editing system to permanent storage takes place and a digital object identifier (DOI) number is assigned to the paper. Once a paper has a unique DOI, it is considered published and is available on the Cureus.com online journal.

The Cureus Scholarly Impact Quotient™ (SIQ™) was examined for each article. The SIQ™ is assigned by Cureus members’ ranking of the article on a ten-point Likert scales [3]. The SIQ™ assigns a point value from 1 to 10 in each of the: clarity of background and rationale, clinical importance, study design and methods, data analysis, the novelty of conclusions, and quality of presentation. The assigned scores are averaged to reach an overall SIQ™ value. The SIQ™ is not displayed until at least two members of the Cureus community have provided their evaluation.

Publication impact metrics for each publication, including the number of times each article was cited and in which journals, were collected using Google Scholar (GS) and Web of Science (WOS) [2,4]. The “latency period” – i.e., the time between publication a given work and the publication of the first article to cite that work – was also analyzed. Our assumption was that a shorter latency period was associated with a higher impact publication. Because some published sources do not provide the exact date of publication, the authors employed a consistent method for estimating the citation’s publication date. In calculating the date of publication when only the month and year were provided, the date was set to the first day of the calendar month. In the one case where only the year was provided, June 1st of that year was used [5].

Statistical analysis

Data were analyzed using descriptive statistics. Numeric data are provided using percentages (%), means, and standard deviations (SD).

## Results

A total of 15 articles by 12 first authors were published on the IPSS Cureus channel during the study period. Ten articles (66%) were original articles (OA), four (27%) were technical reports (TR), and one (7%) was a review article (RA). The complete list of channel publications can be found in Table 1.

**Table 1:** Articles and types of articles published on the IPSS Cureus channel – 2014-2018 OA: original article, RA: review article, TR: technical report, IPSS:  International Pediatric Simulation Society

Published Article	Type
Burns R, Adler M, Mangold K, & Trainor J. (2016). A Brief Boot Camp for 4th-Year Medical Students Entering into Pediatric and Family Medicine Residencies. Cureus, 8(2). Retrieved from https://www.cureus.com/articles/4036-a-brief-boot-camp-for-4th-year-medical-students-entering-into-pediatric-and-family-medicine-residencies [6].	OA
Collins K, Hopkins A, Shilkofski N. A, Levine R. B, & Hernandez R. G. (2018). Difficult Patient Encounters: Assessing Pediatric Residents’ Communication Skills Training Needs. Cureus, 10(9). Retrieved from https://www.cureus.com/articles/13346-difficult-patient-encounters-assessing-pediatric-residents-communication-skills-training-needs [7].	OA
Doherty E, Rachwal C, Lindamood K, O’Brien C, & Weinstock P. (2014). Using Simulated Transport Calls to Identify Diversity of Knowledge and Care Plans among the Multidisciplinary Team. Cureus, 6(10). Retrieved from https://www.cureus.com/articles/2661-using-simulated-transport-calls-to-identify-diversity-of-knowledge-and-care-plans-among-the-multidisciplinary-team [8].	OA
Mackinnon R, Aitken D, & Humphries C. (2015). Exploring Mechanisms for Effective Technology-Enhanced Simulation-based Education in Wilderness Medicine: A Systematic Review. Cureus, 7(12). Retrieved from https://www.cureus.com/articles/3342-exploring-mechanisms-for-effective-technology-enhanced-simulation-based-education-in-wilderness-medicine-a-systematic-review [9].	OA
Mackinnon R, & Gough S. (2014). What Can We Learn About Debriefing From Other High-Risk/High-Stakes Industries? Cureus, 6(4). Retrieved from https://www.cureus.com/articles/2472-what-can-we-learn-about-debriefing-from-other-high-riskhigh-stakes-industries [10].	OA
Mathieson S, Whalen D, & Dubrowski A. (2015). Infant Trauma Management in the Emergency Department: An Emergency Medicine Simulation Exercise. Cureus, 7(9). Retrieved from https://www.cureus.com/articles/2961-infant-trauma-management-in-the-emergency-department-an-emergency-medicine-simulation-exercise [11].	TR
Peacock P. J, Woodman A, Mccay W, & Bates S. E. (2016). Resuscitation of the Newborn: Simulating for Confidence. Cureus, 8(9). Retrieved from https://www.cureus.com/articles/4951-resuscitation-of-the-newborn-simulating-for-confidence. [12].	OA
Roberts J, Sawyer T, Foubare D, Reid J, Stone K, Stephanian D, & Thompson D. (2015). Simulation to Assist in the Selection Process of New Airway Equipment in a Children’s Hospital. Cureus, 7(9). Retrieved from https://www.cureus.com/articles/3154-simulation-to-assist-in-the-selection-process-of-new-airway-equipment-in-a-childrens-hospital [13].	OA
Rosen O, & Angert R. M. (2017). Gastroschisis Simulation Model: Pre-surgical Management Technical Report. Cureus, 9(3). Retrieved from https://www.cureus.com/articles/6225-gastroschisis-simulation-model-pre-surgical-management-technical-report [14].	TR
Sawyer T, Gray M, Hendrickson M, Jacobson E, & Umoren R. (2018). A Real Human Umbilical Cord Simulator Model for Emergency Umbilical Venous Catheter Placement Training. Cureus, 10(11). Retrieved from https://www.cureus.com/articles/15472-a-real-human-umbilical-cord-simulator-model-for-emergency-umbilical-venous-catheter-placement-training [15].	TR
Sawyer T, & Strandjord T. (2014). Simulation-based Procedural Skills Maintenance Training for Neonatal-Perinatal Medicine Faculty. Cureus, 6(4). Retrieved from https://www.cureus.com/articles/2476-simulation-based-procedural-skills-maintenance-training-for-neonatal-perinatal-medicine-faculty [16].	OA
Shea K. L, & Rovera E. J. (2015). Vaginal Examination Simulation Using Citrus Fruit to Simulate Cervical Dilation and Effacement. Cureus, 7(9). Retrieved from https://www.cureus.com/articles/3061-vaginal-examination-simulation-using-citrus-fruit-to-simulate-cervical-dilation-and-effacement [17].	TR
Starr M, Sawyer T, Jones M, Batra M, & Mcphillips H. (2017). A Simulation-based Quality Improvement Approach to Improve Pediatric Resident Competency with Required Procedures. Cureus, 9(6). Retrieved from https://www.cureus.com/articles/6940-a-simulation-based-quality-improvement-approach-to-improve-pediatric-resident-competency-with-required-procedures [18].	OA
Taras J, & Everett T. (2017). Rapid Cycle Deliberate Practice in Medical Education - a Systematic Review. Cureus, 9(4). Retrieved from https://www.cureus.com/articles/6528-rapid-cycle-deliberate-practice-in-medical-education---a-systematic-review [19].	RA
Zaveri P. P, Davis A. B, O’connell K. J, Willner E, Schinasi D. A, & Ottolini M. (2016). Virtual Reality for Pediatric Sedation: A Randomized Controlled Trial Using Simulation. Cureus, 8(2). Retrieved from https://www.cureus.com/articles/3622-virtual-reality-for-pediatric-sedation-a-randomized-controlled-trial-using-simulation [20].	OA

**Figure 1:**
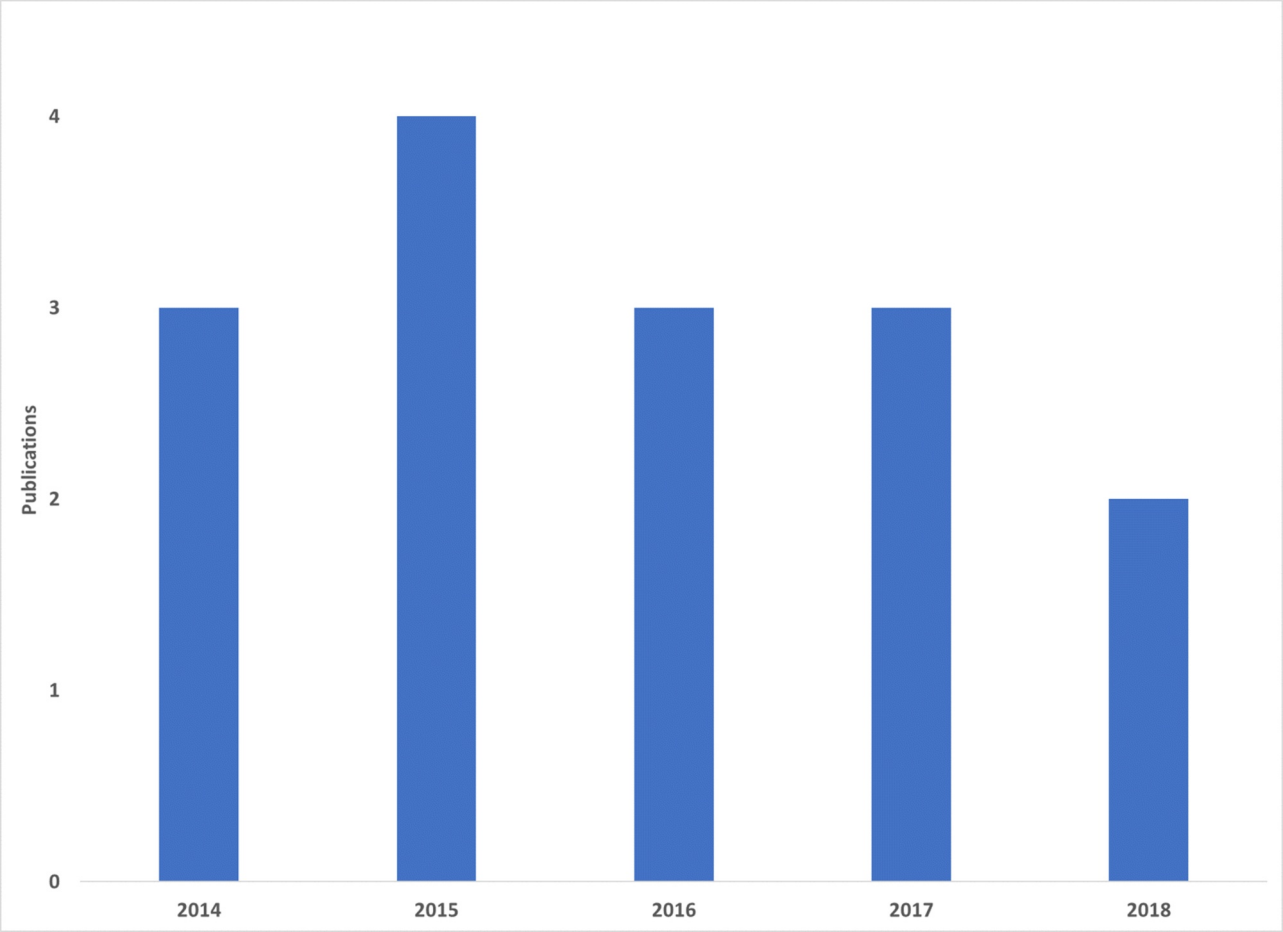
Publications per year on the IPSS Cureus Channel IPSS:  International Pediatric Simulation Society

The number of publications peaked in 2015. See Figure 1. In each case, the authorship was specific to a single country; no group of authors spanned two or more countries. Ten articles were produced by people affiliated to US institutions, three were from the UK, and two were authored by individuals associated with the Canadian universities and medical centers.

Views and downloads

The 15 articles were viewed a total of 11,045 times for an average of 736 views per article (SD: 1102) and downloaded in PDF format a total of 6,690 times, averaging 446 downloads per article (SD: 234) during the period of study. The highest number of views for a single article was 4,270 [16]. The highest number of downloads for a single article was 915 [17].

**Figure 2:**
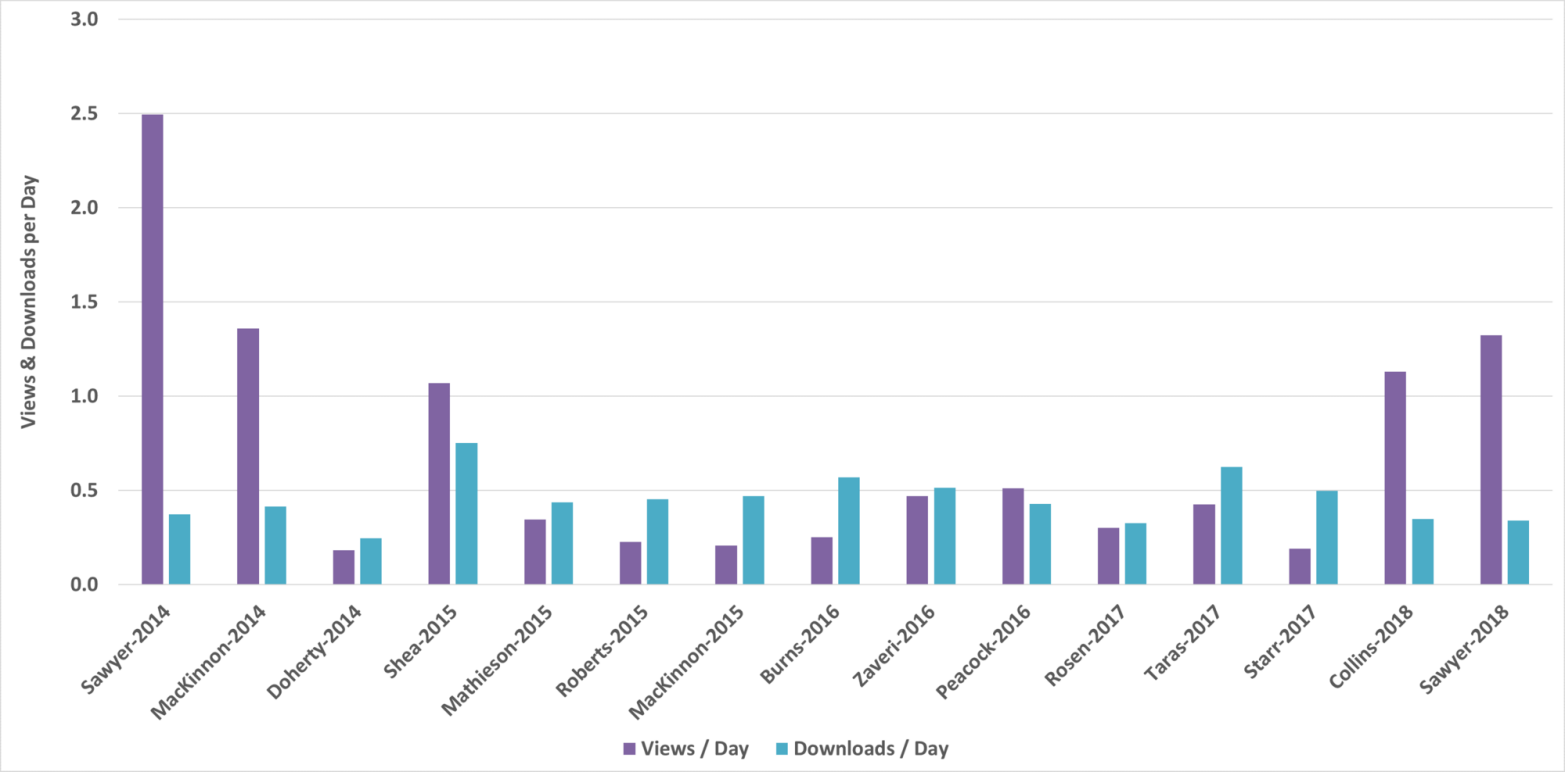
Views per day and downloads per day for each article on the IPSS Cureus channel IPSS:  International Pediatric Simulation Society

The views per day and downloads per day for each article are provided in Figure 2. The mean views per day was 0.7 (SD: 0.6). The mean downloads per day was 0.5 (SD 0.1). The authors further checked if there was a difference in the viewing and download rates between the types of articles published. Because only one RA had been published during the study period, the results are inconclusive, but no appreciable difference was noted between the OA and TR types in either the viewing or downloading rates. See Table 2.

**Table 2:** Views and downloads: descriptive statistics

All Articles	Published	Type	Total Views	Total Downloads	Days Active	Views / Day	Downloads / Day
Sawyer-2014 [16]	4/23/2014	OA	4270	640	1713	2.5	0.4
MacKinnon-2014 [10]	4/25/2014	OA	2324	708	1711	1.4	0.4
Doherty-2014 [8]	10/9/2014	OA	282	384	1544	0.2	0.2
Shea-2015 [17]	9/1/2015	TR	1303	915	1217	1.1	0.8
Mathieson-2015 [11]	9/7/2015	TR	419	529	1211	0.3	0.4
Roberts-2015 [13]	9/24/2015	OA	271	543	1194	0.2	0.5
MacKinnon-2015 [9]	12/17/2015	OA	230	521	1110	0.2	0.5
Burns-2016 [6]	2/9/2016	OA	265	603	1056	0.3	0.6
Zaveri-2016 [20]	2/9/2016	OA	494	542	1056	0.5	0.5
Peacock-2016 [12]	9/20/2016	OA	425	356	832	0.5	0.4
Rosen-2017 [14]	3/22/2017	TR	195	214	649	0.3	0.3
Taras-2017 [19]	4/19/2017	RA	265	389	621	0.4	0.6
Starr-2017 [18]	6/3/2017	OA	110	288	576	0.2	0.5
Collins-2018 [7]	9/21/2018	OA	116	38	101	1.1	0.4
Sawyer-2018 [15]	11/5/2018	TR	76	20	56	1.4	0.4
		Total	11045	6690			
		Mean	736	446	976	0.7	0.5
		SD	1102	234	491	0.6	0.1
		Median	271	521	1056	0.4	0.4
Original Articles	Published	Type	Total Views	Total Downloads	Days Active	Views / Day	Downloads / Day
Sawyer-2014 [16]	4/23/2014	OA	4270	640	1713	2.5	0.4
MacKinnon-2014 [10]	4/25/2014	OA	2324	708	1711	1.4	0.4
Doherty-2014 [8]	10/9/2014	OA	282	384	1544	0.2	0.2
Roberts-2015 [13]	9/24/2015	OA	271	543	1194	0.2	0.5
MacKinnon-2015 [9]	12/17/2015	OA	230	521	1110	0.2	0.5
Burns-2016 [6]	2/9/2016	OA	265	603	1056	0.3	0.6
Zaveri-2016 [20]	2/9/2016	OA	494	542	1056	0.5	0.5
Peacock-2016 [12]	9/20/2016	OA	425	356	832	0.5	0.4
Starr-2017 [18]	6/3/2017	OA	110	288	576	0.2	0.5
Collins-2018 [7]	9/21/2018	OA	116	38	101	1.1	0.4
		Total	8787	4623			
		Mean	879	462	1089	0.7	0.4
		SD	1290	189	480	0.7	0.1
		Median	277	532	1083	0.4	0.4
Technical Reports	Published	Type	Total Views	Total Downloads	Days Active	Views / Day	Downloads / Day
Shea-2015 [17]	9/1/2015	TR	1303	915	1217	1.1	0.8
Mathieson-2015 [11]	9/7/2015	TR	419	529	1211	0.3	0.4
Rosen-2017 [14]	3/22/2017	TR	195	214	649	0.3	0.3
Sawyer-2018 [15]	11/5/2018	TR	76	20	56	1.4	0.4
		Total	1993	1678			
		Mean	498	420	783	0.8	0.5
		SD	481	339	479	0.5	0.2
		Median	307	372	930	0.7	0.4

The publication cycle

**Figure 3:**
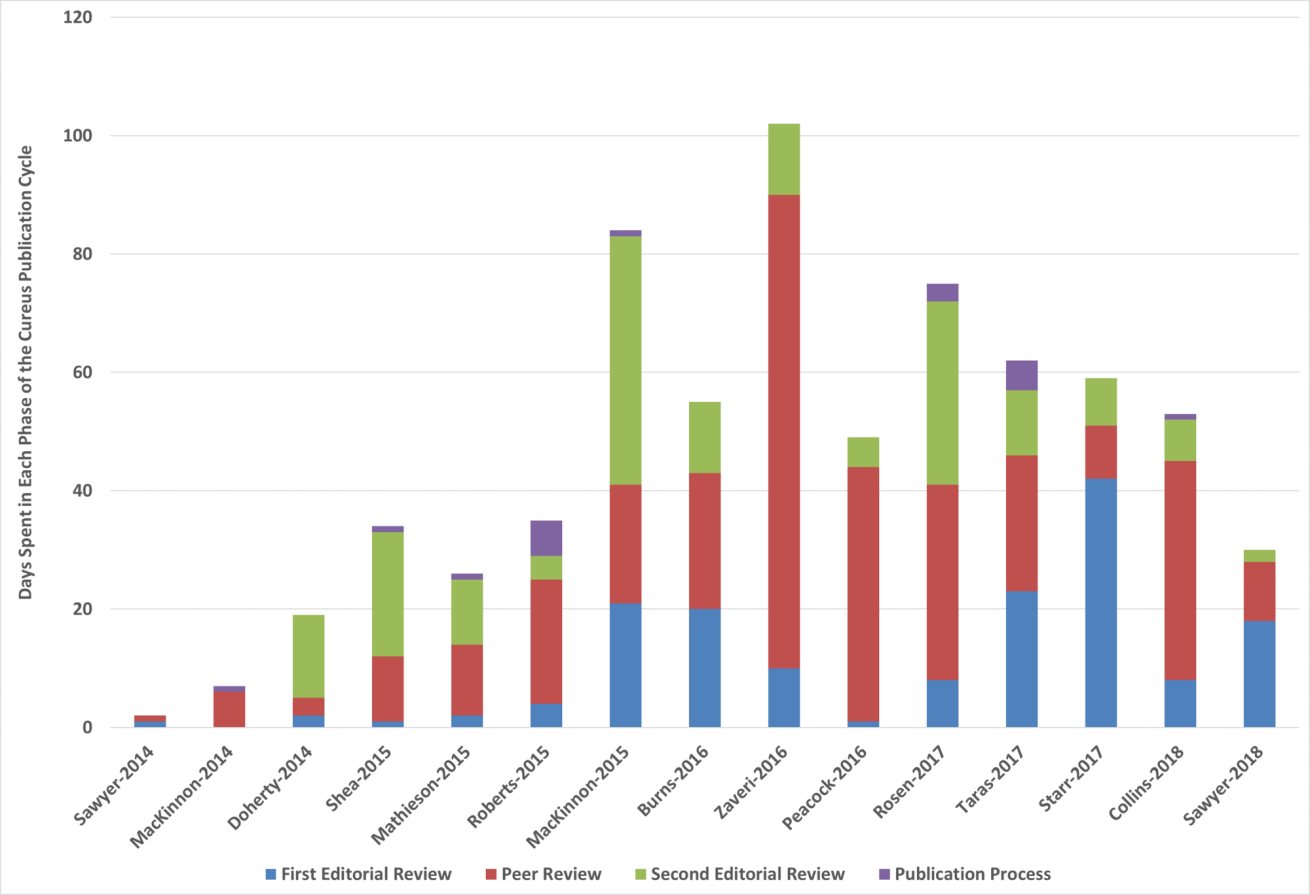
Time spent in each of the four Cureus publication cycle phases

The mean number of days between submission to publication was 46 days (SD: 28). The mean number of days spent in the first editorial review was 11 (SD 12). The mean number of days spent in peer review was 22 (SD: 20). The mean number of days spent in the second editorial review was 12 (SD: 12). The mean number of days spent in the publication process was one (SD: 2). A graph showing the time each article spent in each of the phases can be found in Figure 3. On average, the peer review phase accounted for 48% of the publication process.

Scholarly impact quotient™

Of the 15 articles, only six have achieved the required two rankings to receive a SIQ™ [10,11,13,16,19-20]. Only one had four rankings [13]. All six of the ranked articles were rated above the midpoint score of 5.0 on the ten-point scale, with a mean score of 6.7 (SD: 0.8).

Impact analysis

**Figure 4:**
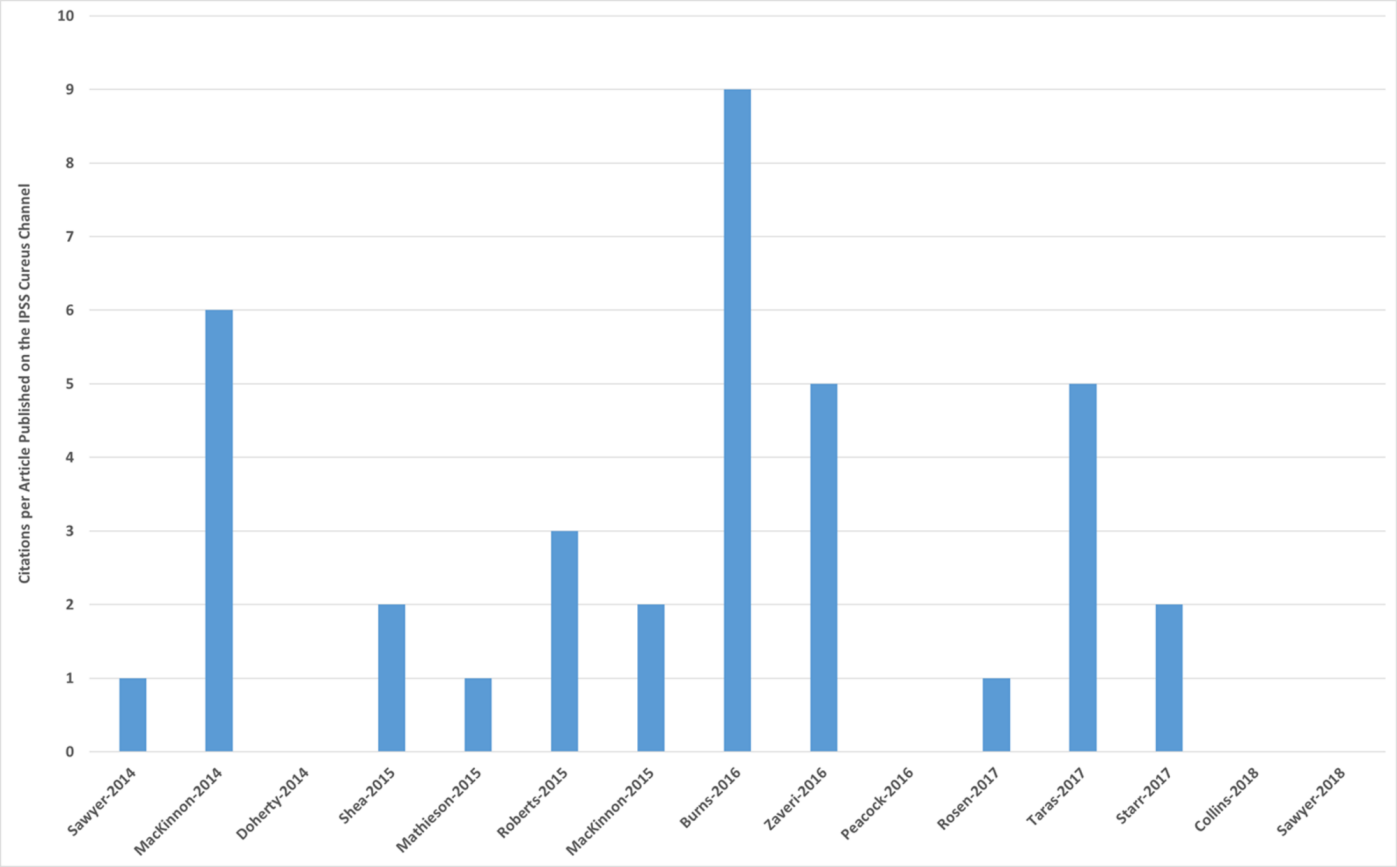
Citation counts for articles published on the IPSS Cureus channel

The 15 articles were cited a total of 36 times (average 2.5 citations per article). The citations are presented in Table 3, and the distribution of citations is shown in Figure 4.

The global reach of the IPSS Cureus channel can be estimated from the countries where the citations originated, and the languages used in the cited articles. Table 3 shows that while most of the citing sources originated in the United States and English was the predominant language, four of the citing articles were written in Spanish, two in Portuguese, and one each in Arabic and Ukrainian.

**Table 3:** Source publications where citations appeared, home country of publication, and original language

Published In / By	Country	Language
7th International Conference on Information, Intelligence, Systems Applications (IISA)	USA	English
Academic Pediatrics	USA	English
Advances in Simulation	Switzerland	English
American Heart Association	USA	English
American Journal of Medical Quality	USA	English
Annals of Emergency Medicine	USA	English
ARS MEDICA Revista de Ciencias Médicas	Chile	Spanish
Australasian Emergency Care	Australia	English
BMJ Simulation and Technology Enhanced Learning	UK	English
Clinical Pediatric Emergency Medicine	UK	English
Comprehensive Healthcare Simulation: Pediatrics (Book)	Switzerland	English
Cureus	USA	English
Doctoral dissertation	UK	English
Dominican Republic: Ministry of Public Health (Written Protocol for Care)	Dominican Republic	Spanish
Healthcare Simulation Education Evidence: Theory and Practice. (Book)	UK	English
International Journal of Radiation Oncology*Biology*Physics	The Netherlands	English
Jornal de Pediatria	Brazil	Portuguese
Journal for Nurses in Professional Development	USA	English
Journal of Medical Imaging and Radiation Sciences	The Netherlands	English
Journal of Pediatric Surgery	USA	English
Medicni perspektivi (Medical Perspectives)	Ukraine	Ukrainian
Medical Science Educator	USA	English
Midwifery	USA	English
Otolaryngologic Clinics of North America	UK	English
Paediatrics & Child Health	Canada	English
Proceedings of the 1st International Conference: New Perspectives in Electrical & Computer Engineering	Morocco	Arabic
Revista Española de Anestesiología y Reanimación	Spain	Spanish
Revista Española de Pediatría	Spain	Spanish
Revista Multidisciplinar e de Psicologia	Brazil	Portuguese
Seminars in Fetal and Neonatal Medicine	The Netherlands	English
Seminars in Perinatology	UK	English
Simulation in Healthcare	USA	English
Society for Teachers of Family Medicine Spring Conference (Poster)	USA	English
Southern Medical Journal	USA	English
Technology and Health Care	The Netherlands	English

**Figure 5:**
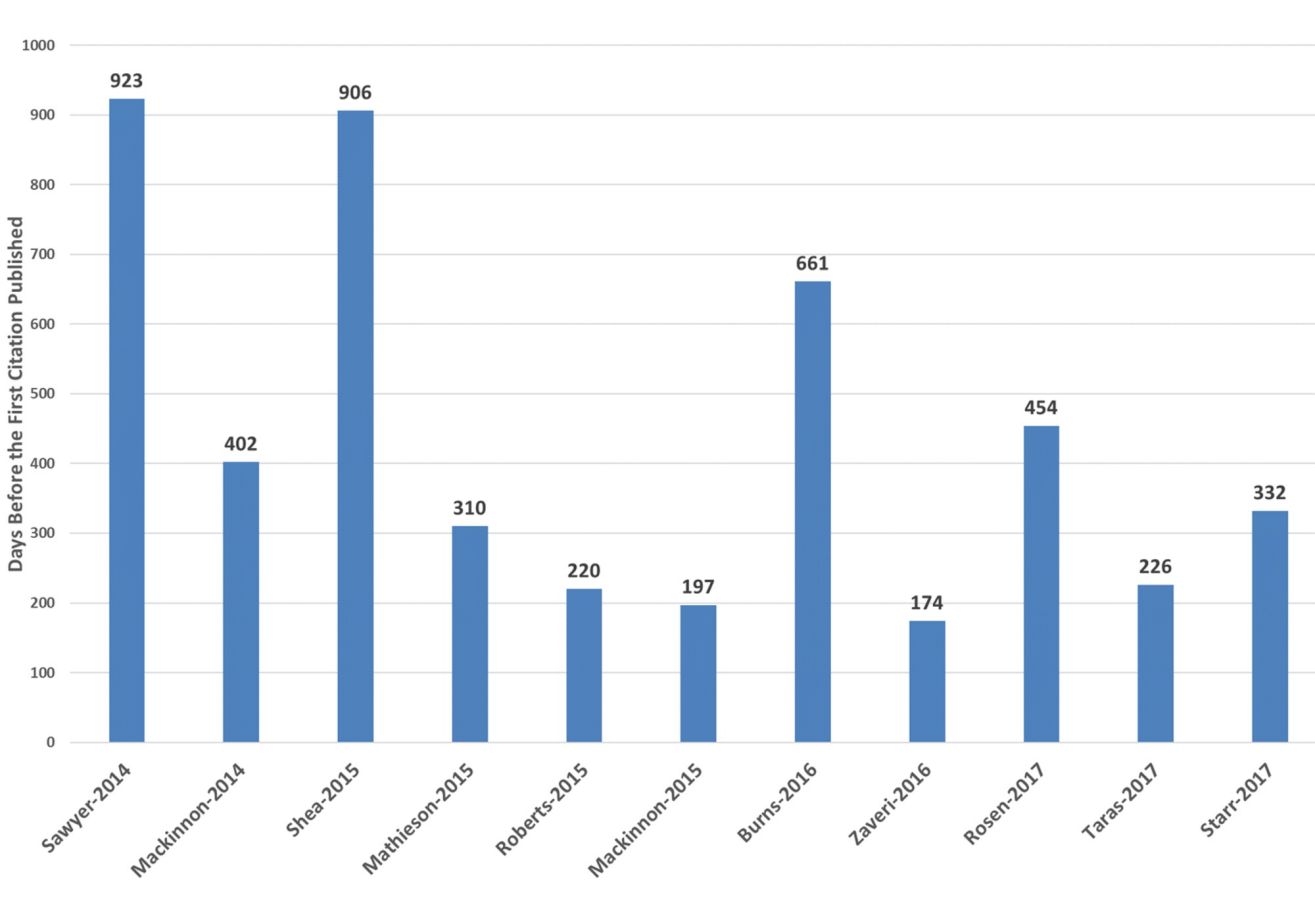
Citation latency period for each cited article on the IPSS Cureus channel IPSS:  International Pediatric Simulation Society

Figure 5 shows the latency periods for each article cited. The mean latency period for the 11 articles cited was 437 days (SD: 274 days). The longest latency period was 923 days, while the shortest latency period was 174 days [16,20].

## Discussion

Our analysis shows an overall low level of publication activity on the IPSS Cureus channel with only 15 publications over 55 months. Publications on the channel have decreased over time, further indicating that the channel is underused by IPSS members. Published articles, however, appear to have a large impact regarding views, downloads, and citations. TRs compare favorably to original articles in terms of views and downloads, being viewed and downloaded slightly more than original articles. Increased member awareness may increase article submissions. We hope that providing this detailed analysis of the impact of articles on the IPSS Cureus channel will foster more interest in using the channel by IPSS members.

Because a relatively small number of articles have been published on the IPSS Cureus channel over the past 55 months, performing anything beyond a descriptive analysis was not possible. However, we feel our analysis uncovered some interesting points. First, 11 of the articles had a wide impact, both in terms of the journals and other publications that cited them, but also through the authorship of the articles citing them. All the articles published on the channel were authored by groups within the same country and mostly within the same institutions. The sources that cited them, however, showed a far greater international variety. Six of the 37 citations to IPSS Cureus channel publications were authored by international teams. Eight sources were originally published in languages other than English from countries ranging from Spain, Chile, and the Dominican Republic (Spanish) to Brazil (Portuguese) to Morocco (Arabic) and Ukraine (Ukrainian). In one case, a citation to an IPSS Cureus channel publication was from a protocol on the treatment of gastroschisis, a rare birth defect, issued by the Ministry of Public Health of the Dominican Republic [21].

The statistics on views and download rates per day presented some noteworthy anomalies. The most viewed article was the first one published on the channel [16]. This would be expected since it has been available longer than any other article, but the views per day (2.5) were almost twice that of the next highest number of views per day (1.4) [10,15]. The authors are unable to fully explain this phenomenon.

The most downloaded article was a TR from 2015 with 915 downloads during the study period or a mean of 0.8 downloads per day [17]. This was 25% more than the next highest number of downloads per day of 0.6 [6,19]. The high rate of download may be partially explained by the nature of the TR itself, which described a simple, inexpensive method of simulating vaginal dilation and effacement. That type of TR may have attracted significant attention in low resource areas of the world. Unfortunately, the authors had no data on where the downloads originated, and hence, they could not test this hypothesis.

This study has several limitations. The lack of data on where views and downloads originate limits the ability to measure the overall global impact of the publications on the IPSS Cureus channel. Other limitations of this study include the lack of any comparison group of articles from outside the channel and the small population of articles being studied. Finally, the lack of data on views and downloads over time limits the ability to make inferences regarding the sustained interest in each article.

## Conclusions

Our analysis of the IPSS Cureus channel publication activity shows that publications on the channel have a clear impact. The channel offers a rapid submission-to-publication cycle with an average publication turn-around of less than two months, faster than most other peer-reviewed medical journals.

The next step in the analysis of the IPSS Cureus channel will be to determine a reference collection of Cureus articles to use as a control group and then reevaluate the impact of the channel against that of the comparison group. A complete bibliometric examination of the Cureus journal might also show interesting trends and present further support for the Cureus model of medical information dissemination.
